# Integrated single-cell and transcriptomic profiling identifies machine-learning–based pyroptosis biomarkers in IBD

**DOI:** 10.3389/fimmu.2026.1761476

**Published:** 2026-02-04

**Authors:** Yao Lu, Yahui Lin, Yuansen Li, Huan Sai, Cheng Chen, Jinjiao Li

**Affiliations:** 1 Department of Gastroenterology, The People’s Hospital of Wenshan Prefecture, Wenshan, Yunnan, China; 2 Department of Gastrointestinal and Hernia Surgery, The First Affiliated Hospital of Kunming Medical University, Kunming, Yunnan, China; 3 Department of Breast, Thyroid and Burn Surgery, The People’s Hospital of Wenshan Prefecture, Wenshan, Yunnan, China

**Keywords:** inflammatory bowel disease, machine learning, pyroptosis, single-cell RNA sequencing, transcriptomic integration

## Abstract

**Background:**

Pyroptosis, an inflammatory form of programmed cell death, contributes to intestinal inflammation in inflammatory bowel disease (IBD), but the key cell types and regulatory genes remain unclear.

**Methods:**

We analyzed single-cell RNA-seq data from intestinal mucosa to assess pyroptosis-related gene expression signatures using AUCell, AddModuleScore, and GSVA. High- and low-pyroptosis cells were compared to identify core genes. Findings were validated in bulk peripheral blood transcriptomic data. Machine learning (LASSO, GBM, SVM, Boruta, Random Forest) identified optimal diagnostic genes, and functional enrichment explored biological roles.

**Results:**

Pyroptosis was most active in macrophages, epithelial cells, and neutrophils. Six cell subsets consistently exhibited high pyroptosis. Nineteen core pyroptosis-related genes were identified, 16 of which were upregulated in both mucosal and blood samples. Functional analysis linked these genes to apoptosis and reactive oxygen species pathways. Machine learning highlighted six key diagnostic genes—BASP1, LITAF, NAMPT, PHACTR1, PLAUR, PPIF—with BASP1 showing the strongest performance (AUC = 0.935).

**Conclusions:**

Pyroptosis is highly active in specific immune and epithelial cells in IBD. Six identified genes show potential as non-invasive diagnostic biomarkers, offering insights into disease mechanisms and therapeutic targets.

## Introduction

Inflammatory bowel disease (IBD) has emerged as a chronic disorder with a steadily increasing global incidence, placing a substantial and ongoing burden on healthcare systems and patient quality of life (1). IBD, comprising ulcerative colitis (UC) and Crohn’s disease (CD), is characterized by chronic, relapsing intestinal inflammation and epithelial damage. Its pathogenesis is complex (2), involving genetic susceptibility, environmental triggers, gut microbiota dysbiosis, and immune dysregulation. Pyroptosis, a pro-inflammatory form of programmed cell death mediated by Gasdermin family proteins, plays a key role in regulating mucosal innate immune responses and intestinal pathogen defense. By activating multiple inflammatory pathways through damage-associated signaling, pyroptosis may contribute to chronic intestinal inflammation (3). Nevertheless, the specific cellular mechanisms and genetic factors that govern pyroptosis in IBD remain largely unexplored (4). Investigating pyroptosis within intestinal immune and epithelial cells could provide novel insights into IBD pathogenesis and offer potential avenues for improved diagnosis and therapeutic intervention (4–6).

Recent advances in single-cell RNA sequencing (scRNA-seq) have enabled high-resolution dissection of the intestinal mucosa, allowing the identification of heterogeneous cell populations and their transcriptional states (7). This technology provides opportunities to uncover cell-type-specific alterations and molecular programs underlying IBD pathogenesis. Integrating scRNA-seq with bulk transcriptomic data further facilitates the validation of key molecular features across local (mucosal) and systemic (peripheral blood) compartments, offering robust biomarkers with potential clinical relevance (8). In addition, machine learning–based feature selection provides a powerful strategy to identify optimal diagnostic genes from complex multi-omics datasets (9).

In this study, we systematically profiled pyroptosis-related activity across intestinal mucosal cell populations in IBD using single-cell RNA sequencing, and explored potential pyroptosis-associated regulatory genes. These candidates were further examined using bulk transcriptomic datasets. In addition, multiple machine learning approaches were applied to refine and prioritize a minimal set of putative diagnostic feature genes. Collectively, these analyses may provide additional perspectives on the molecular regulation of IBD and offer potential clues for biomarker development in disease diagnosis and therapeutic decision-making.

## Methods

### Data acquisition and processing

The single-cell RNA sequencing (scRNA-seq) data used in this study were obtained from the GSE214695 dataset, which includes samples from 6 patients with UC, 6 patients with CD, and 6 healthy controls (HC) (10). Based on previous studies (3, 4), we selected 10 pyroptosis-related genes associated with the canonical inflammasome pathway (NLRP1, NLRP3, NLRC4, AIM2, Pyrin, PYCARD, CASP1, IL1B, IL18, and GSDMD). After quality control, cells with fewer than 200 or more than 2,500 detected genes or with a mitochondrial gene proportion exceeding 5% were excluded, resulting in high-quality cells for downstream analysis. The top 2,000 highly variable genes were identified using the “vst” method to provide a stable basis for subsequent dimensionality reduction and clustering (11). To mitigate potential technical variation across samples, batch effects were corrected using the Harmony algorithm implemented in Seurat, using principal components derived from PCA with default parameters (theta = 2, lambda = 1) (12). Cells were clustered using Seurat’s graph-based clustering approach with a resolution parameter set to 0.5. Based on the batch-corrected data, unsupervised clustering was performed using the Seurat package with a resolution of 0.5, and UMAP was applied for dimensionality reduction and visualization, revealing diverse intestinal mucosal cell subpopulations (13). Cell annotation was conducted by integrating the original nanostring_reference information with known cell marker genes, ultimately identifying 54 distinct cell subpopulations based on the nanostring_reference for downstream analysis, encompassing various cell types (10). For bulk RNA-seq data, external validation was performed using peripheral blood bulk transcriptome data (GSE3365) (14).

### Gene-set scoring and correlation analysis in scRNA-seq

pyroptosis-related gene expression signatures was quantified using AUCell (15), AddModuleScore (16), and GSVA (17) based on a curated pyroptosis gene set. AUCell calculated the AUC of ranked gene expression per cell; AddModuleScore generated normalized module scores; GSVA estimated pathway-level enrichment for each cell or cluster. Cells were divided into high- and low-pyroptosis groups according to score distribution. Differential expression was analyzed using Seurat’s *FindMarkers* (Wilcoxon test). Gene–pyroptosis associations were assessed using Spearman correlation between gene expression and pyroptosis scores across cells or clusters. Subsequently, Gene Ontology (GO) enrichment analysis was performed using the clusterProfiler R package to explore the potential biological mechanisms associated with these differentially expressed genes.

### Machine learning-based identification of optimal diagnostic genes

To evaluate the diagnostic potential of candidate genes identified from single-cell analysis, we applied an integrative machine learning–based feature selection framework to an independent peripheral blood transcriptomic dataset. Gene expression normalization and feature selection were conducted on the complete peripheral blood dataset in the context of gene prioritization.

To identify the most robust diagnostic markers, we integrated five machine learning algorithms, including LASSO (18), GBM (19), SVM-RFE (20), Boruta (21), and random forest (22). To ensure reproducibility, all machine learning analyses were performed using a fixed random seed (set.seed(123)). LASSO regression was implemented using the *glmnet* package, in which an L1 penalty was applied to shrink coefficients and eliminate redundant variables, with 10-fold external validation used to determine the optimal regularization parameter (λ). Gradient boosting machine (GBM) models were constructed using default parameters (n.trees = 100, interaction.depth = 1, shrinkage = 0.1), and random forest models were built with default settings (including ntree = 500), to rank feature importance based on ensemble learning and thereby improve the stability of feature selection. SVM-RFE was used to iteratively remove the least informative genes and retain the most discriminative features, and was implemented using a linear kernel with default settings. In parallel, the Boruta algorithm was employed to identify all relevant variables by comparing their importance with that of randomly permuted shadow features, and was applied using default parameters (maxRuns = 100, pValue = 0.01), thereby ensuring comprehensive feature extraction. Finally, the intersecting genes identified by all five algorithms were defined as the optimal diagnostic gene set for further analysis.

Rather than constructing a standalone predictive classifier, these machine learning methods were applied primarily for robust feature selection and ranking. Genes consistently identified by all five algorithms were defined as the final diagnostic gene set. ROC analyses were performed using the same peripheral blood transcriptomic dataset employed for feature selection, without a separate held-out testing set, with the primary objective of gene prioritization.

### Statistical analysis

All statistical analyses were conducted in R (version 4.4.0). Group differences were assessed using Student’s *t*-test or Wilcoxon rank-sum test as appropriate, and multiple-group comparisons used one-way ANOVA or Kruskal–Wallis tests. Correlations between pyroptosis scores and gene expression were evaluated using Spearman’s correlation. Differentially expressed genes were identified with Benjamini–Hochberg FDR–adjusted *P* values < 0.05. A two-sided *P* < 0.05 was considered statistically significant.

## Result

### Single-cell RNA-seq analysis of IBD

**Figure 1:**
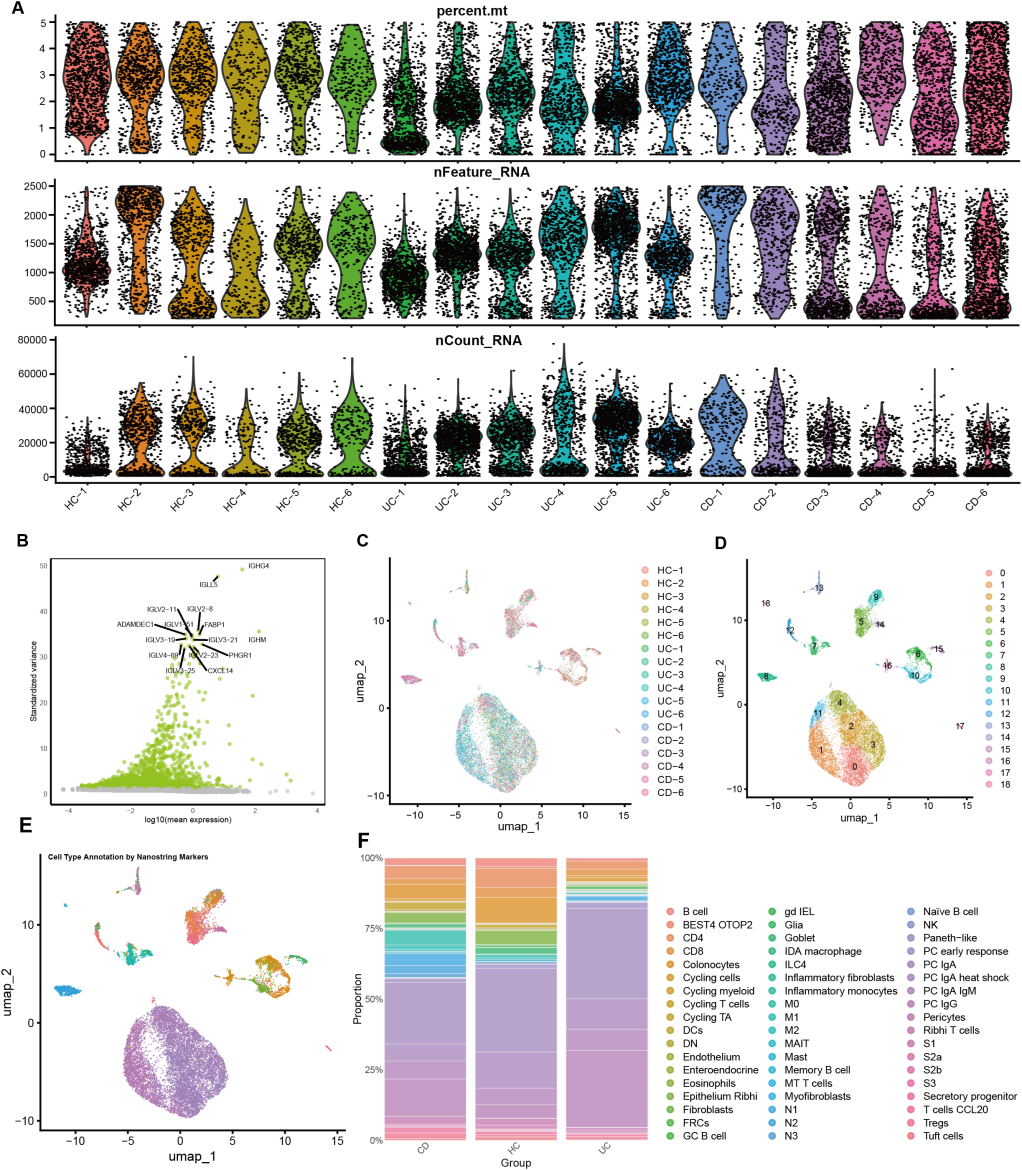
Cellular subpopulation landscape and characteristic analysis. **(A)** Quality control of single-cell RNA-sequencing data. **(B)** Identification of highly variable genes. **(C)** Batch effect correction. **(D)** UMAP dimensionality reduction and clustering based on the Seurat algorithm, illustrating the distribution and classification of intestinal mucosal cells. **(E)** Cell annotation revealed 54 distinct cellular subpopulations, including immune and epithelial cell types. **(F)** Proportional distribution of each cellular subpopulation across healthy controls, ulcerative colitis, and Crohn’s disease groups.

Prior to downstream analyses, we performed stringent quality control and selected highly variable genes, resulting in a high-quality dataset (**Figures 1A, B**). Batch effects across samples were then corrected, leading to a more stable distribution (**Figure 1C**). Following the standard Seurat workflow, we conducted clustering and identified 18 distinct cell subpopulations (**Figure 1D**). Given that the GSE214695 dataset provides high-quality cell-type annotations that are highly consistent with our clustering results, all subsequent analyses were based on the original annotations (**Figure 1E**). Comparing cell compositions across different pathological states revealed substantial differences among healthy controls, ulcerative colitis, and Crohn’s disease (**Figure 1F**). Notably, several immune cell subsets were markedly increased in inflammatory conditions, suggesting that these cells may play key roles in the pathogenesis of IBD.

### Pyroptosis in single-cell RNA-seq data

**Figure 2:**
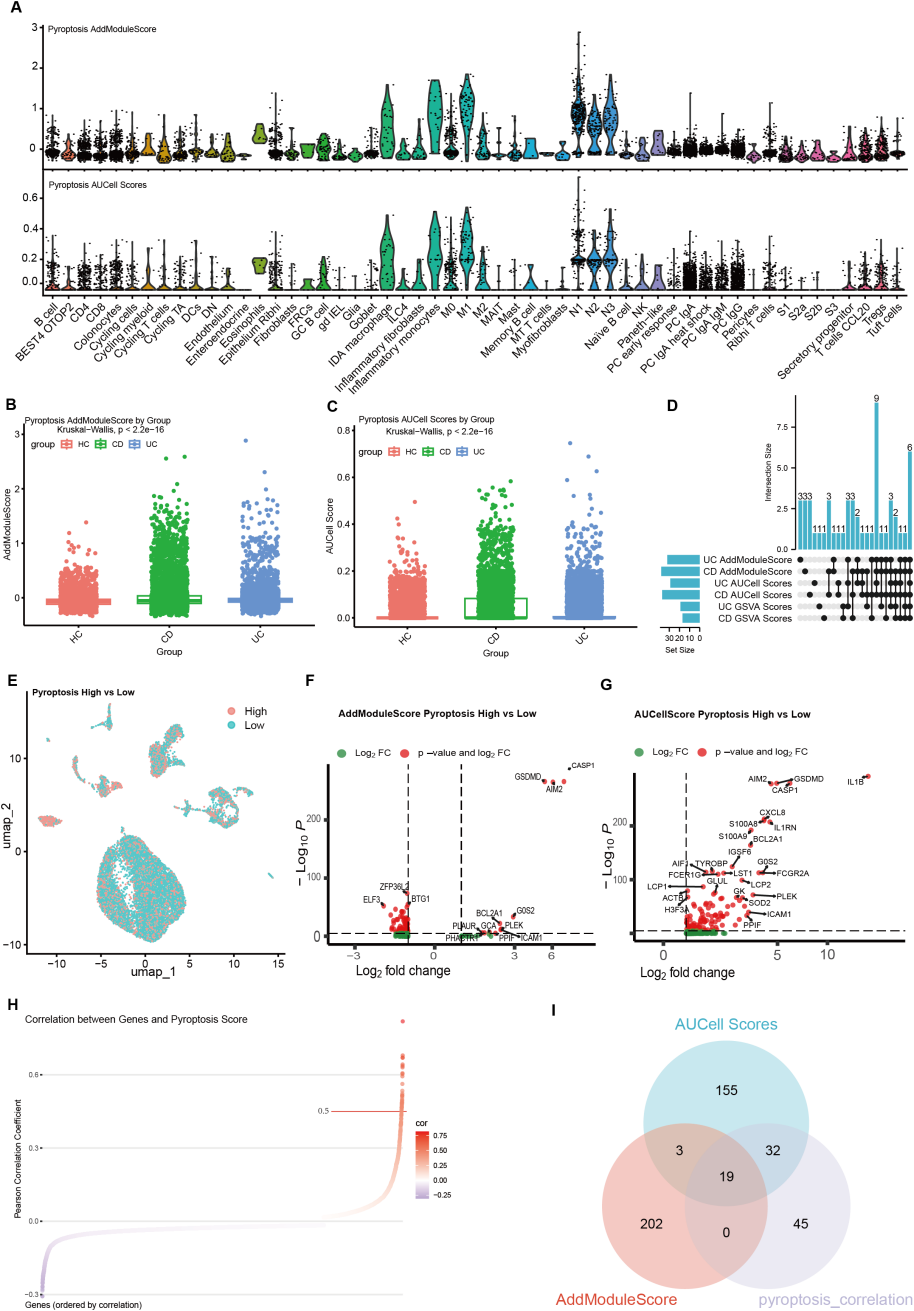
Pyroptosis scoring and differential analysis in single-cell data. **(A)** Pyroptosis-related gene set scores calculated across different cell types using the AUCell and AddModuleScore algorithms. **(B, C)** Differences in pyroptosis-related gene expression signatures scores between healthy controls (HC) and inflammatory bowel disease (IBD) groups (B: AddModuleScore; C: AUCell). **(D)** Cell types showing consistently elevated pyroptosis scores across AUCell, GSEA, and AddModuleScore. **(E)** Cells stratified into high- and low-pyroptosis groups based on pyroptosis scores. **(F, G)** Percentage changes and log-fold changes of differentially expressed genes between high- and low-pyroptosis groups (F: AddModuleScore; G: AUCell). **(H)** Correlation analysis between pyroptosis scores and the overall expression of pyroptosis-related genes. **(I)** Venn diagram identifying key genes closely associated with pyroptosis.

**Figure 3:**
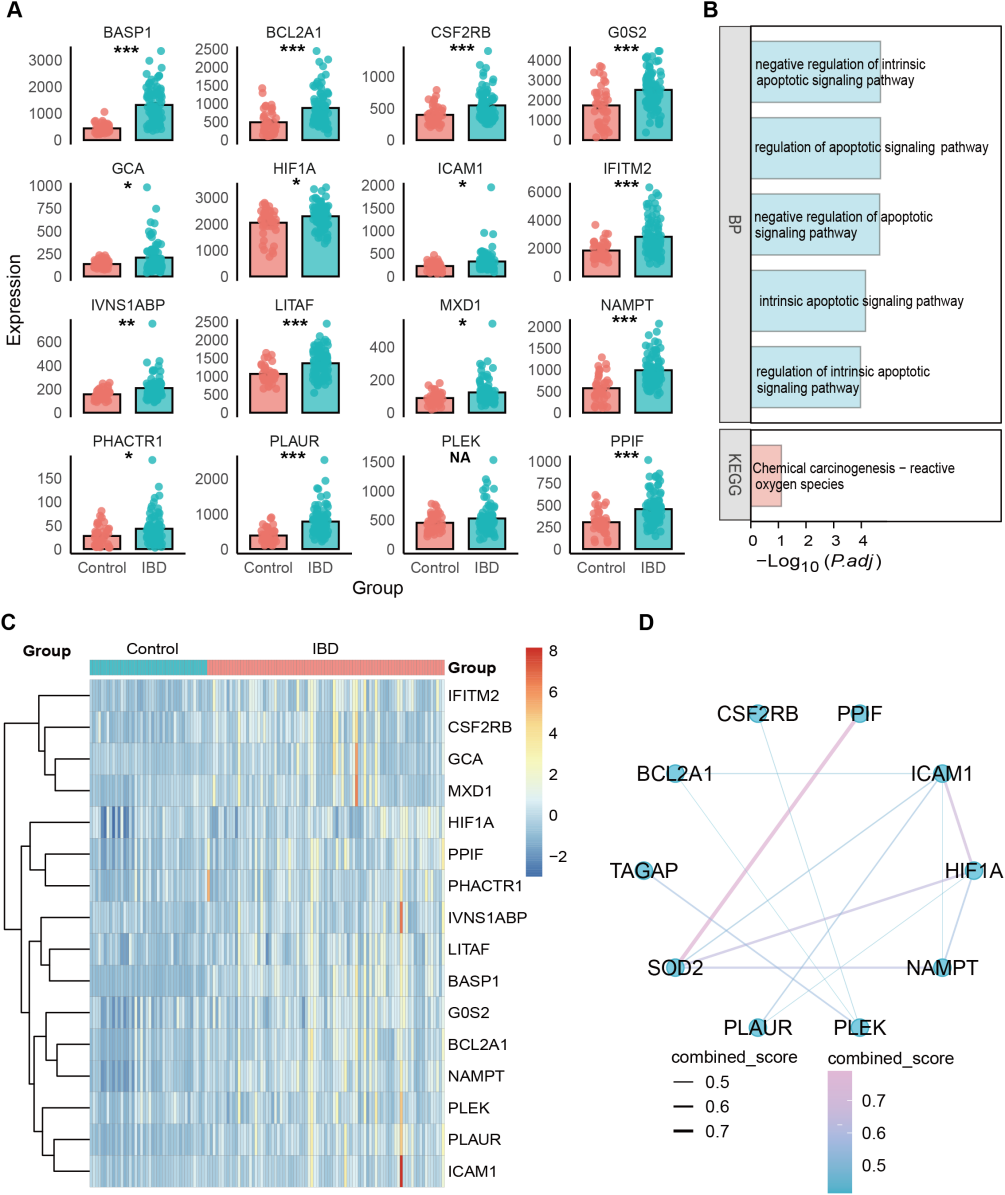
Cross-analysis of key genes based on bulk RNA-seq data. **(A)** Differential expression of the 16 overlapping genes identified from scRNA-seq and bulk transcriptomic datasets. **(B)** GO and KEGG enrichment analysis results. **(C)** Heatmap showing the expression patterns of these 16 genes in control and IBD samples. **(D)** Gene interaction network.

We evaluated the expression of pyroptosis-related gene sets at the single-cell level using the AUCell and AddModuleScore algorithms. pyroptosis-related gene expression signatures exhibited substantial heterogeneity across different cell types; specifically, these genes were more highly enriched in macrophages, intestinal epithelial cells, and neutrophils (**Figure 2A**). Both the Crohn’s disease and ulcerative colitis groups showed higher overall pyroptosis scores than healthy controls (HC) across multiple immune and epithelial cell populations (**Figures 3B, C**). Using AUCell, AddModuleScore, and GSVA, we assessed pyroptosis-related gene expression signatures across the three groups within each cell type (**Supplementary Material 1**). Across all three scoring methods, six cell populations consistently exhibited higher pyroptosis-related gene expression in both the UC and CD groups: colonocytes, inflammatory monocytes, N1, N2, N3, and M1. By stratifying cells into high- and low-pyroptosis groups according to average expression levels, we further demonstrated that these genes were predominantly expressed within the neutrophil clusters (**Figure 2E**).

To identify genes associated with pyroptosis-related gene expression signatures, we categorized cells into high-pyroptosis and low-pyroptosis groups based on the AUCell and AddModuleScore pyroptosis scores, respectively. Differential expression analysis between the two groups was performed using Seurat’s *FindMarkers* function (Wilcoxon rank-sum test). Using the AUCell-derived scores, we identified 118 upregulated genes, whereas the AddModuleScore-based scoring yielded 14 upregulated and 140 downregulated genes (FDR < 0.05; FindMarkers criteria: |log2FC| > 1, min.pct > 0.1). To further determine genes most closely associated with pyroptosis, we conducted correlation analysis and identified 96 genes significantly correlated with pyroptosis-related gene expression signatures (r > 0.3, FDR < 0.05) (**Figure 2H**). To avoid over-filtering and potentially missing important candidates, all DEGs identified by either scoring method (FDR < 0.05, |log2FC| > 0) were retained for constructing the intersections shown in **Figure 2I**. The intersection between the correlated genes and the differentially expressed genes resulted in 19 pyroptosis-associated genes (**Figure 2I**).

### Cross-analysis of overlapping genes based on bulk transcriptomic data

Given that IBD is characterized by both localized intestinal mucosal inflammation and systemic immune activation, we performed external validation by evaluating candidate genes identified from intestinal mucosal scRNA-seq analysis in an independent peripheral blood bulk transcriptomic dataset (GSE3365). Although the two datasets originate from different biological sources, the consistency of key gene expression patterns at both local (mucosa) and systemic (peripheral blood) levels would further support their stable and broad biological relevance in IBD. To verify the reliability of the 19 selected genes, we further evaluated their expression profiles in the bulk transcriptome dataset. Cross-comparison between scRNA-seq–identified genes and bulk data revealed 16 overlapping genes. All 16 genes were significantly upregulated in the IBD group (**Figure 3A**). GO and KEGG enrichment analyses indicated that these genes were primarily involved in the regulation of intrinsic and overall apoptotic signaling pathways and were closely associated with reactive oxygen species–mediated chemical carcinogenesis (**Figure 3B**). A heatmap further supported these findings, showing a consistent high-expression pattern of the 16 genes in the IBD samples (**Figure 3C**). Among these upregulated genes, SOD2, PPIF, HIF1A, NAMPT, ICAM1, and PLEK exhibited higher DC scores and formed a tightly connected interaction network with the other genes (**Figure 3D**).

### Identification of optimal genes using machine learning

**Figure 4:**
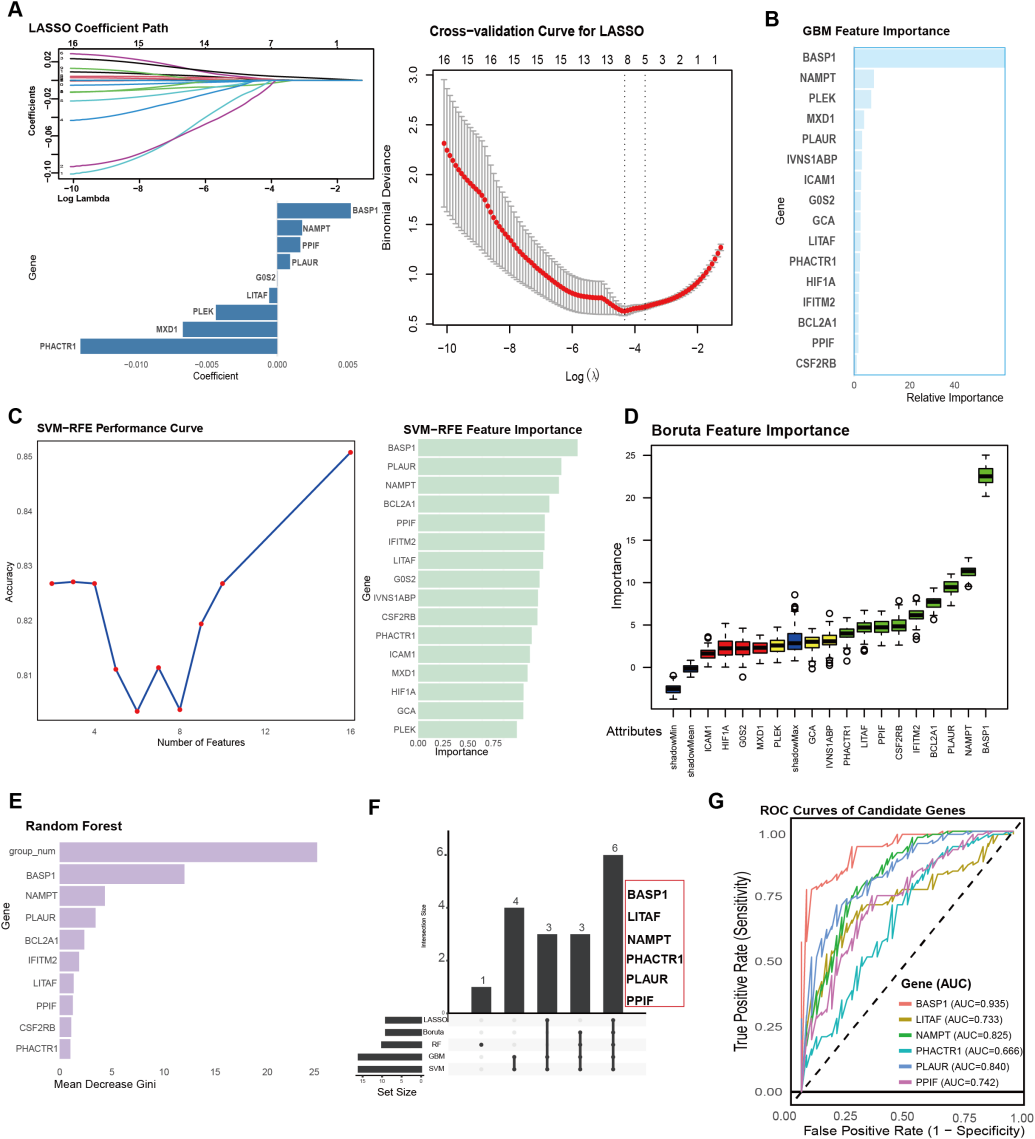
Identification and diagnostic evaluation of optimal feature genes by machine learning. **(A–E)** Feature gene selection using five machine learning algorithms: LASSO **(A)**, GBM **(B)**, SVM **(C)**, Boruta **(D)**, and Random Forest **(E)**. Each algorithm identifies candidate genes with predictive relevance. **(F)** Intersection analysis of the genes selected by all five algorithms. **(G)** Receiver operating characteristic (ROC) curve analysis evaluating the diagnostic performance of the six genes in peripheral blood.

We applied five machine learning algorithms to screen for signature genes, including the LASSO algorithm (**Figure 4A**), GBM algorithm (**Figure 4B**), SVM-RFE algorithm (**Figure 4C**), Boruta algorithm (**Figure 4D**), and random forest model (**Figure 4E**). Intersection analysis of the marker genes identified by all five algorithms yielded six characteristic genes: BASP1, LITAF, NAMPT, PHACTR1, PLAUR, and PPIF (**Figure 4F**). We further evaluated the diagnostic performance of these six genes in peripheral blood. ROC curve analysis (**Figure 4G**) showed that all six genes exhibited good diagnostic value, with BASP1 displaying the strongest diagnostic ability (AUC = 0.935), followed by PLAUR (AUC = 0.840), NAMPT (AUC = 0.825), PPIF (AUC = 0.742), LITAF (AUC = 0.733), and PHACTR1 (AUC = 0.666).

## Discussion

Pyroptosis, as a highly inflammatory form of programmed cell death, plays a pivotal role in the onset and progression of IBD. Numerous studies have shown that pyroptosis not only disrupts intestinal epithelial barrier integrity but also regulates the activation states of multiple immune cell populations, thereby amplifying intestinal inflammatory cascades (4, 23). Thus, pyroptosis represents a critical entry point for understanding the immunopathogenesis of IBD and offers a promising therapeutic target for precision intervention (24).

By integrating AUCell, AddModuleScore, and GSVA scoring methods, we identified six immune and epithelial subpopulations (colonocytes, inflammatory monocytes, N1, N2, N3, and M1) that consistently exhibited elevated pyroptosis-related gene expression signatures. Differential expression and correlation analyses further revealed a set of 19 core pyroptosis-associated genes, which were strongly supported by bulk transcriptomic data from peripheral blood. Sixteen genes overlapped between mucosal and systemic datasets, underscoring their potential as stable biomarkers and highlighting the systemic consequences of local intestinal inflammation. Functional enrichment analyses indicated that these genes are primarily involved in apoptotic regulation and reactive oxygen species–mediated processes, suggesting mechanistic links among pyroptosis, oxidative stress, and epithelial injury.

Notably, Machine learning identified six diagnostic genes (BASP1, LITAF, NAMPT, PHACTR1, PLAUR, and PPIF) with strong predictive performance in peripheral blood. BASP1 showed the highest diagnostic accuracy and may represent a key gene associated with pyroptosis-related inflammation. By integrating intestinal mucosal single-cell data with peripheral blood bulk transcriptomes, our multi-cohort analysis consistently supported BASP1 as an IBD-associated molecule linked to pyroptosis-related gene expression signatures, providing the first evidence for this potential connection. Previous studies have linked BASP1 to IBD through genetic susceptibility (25) and immune infiltration (26) analyses, including the Immunochip meta-analysis and WGCNA-based UC study, yet its role in pyroptosis remains unexplored.

The remaining five genes differ in their biological relevance to pyroptosis, representing distinct modules involving metabolism, mitochondrial stress, and inflammatory transcriptional regulation. PLAUR, a classical mediator of inflammation and tissue remodeling, is upregulated in IBD mucosa and in cytokine-induced epithelial barrier disruption (27), serving as an important marker of epithelial hypoxia responses and mucosal repair in IBD (28). Although PLAUR may interact with IL-1β production and inflammasome activation (29), its influence on pyroptosis appears to be indirect—primarily enhancing inflammatory signaling rather than functioning as a canonical GSDM-dependent pyroptosis gene. NAMPT, a dual mediator of inflammation and metabolism, is markedly elevated in IBD, reflecting both inflammatory burden and tissue hypoxia (30), and correlates strongly with responses to anti-TNF therapy (31); moreover, neutralizing extracellular NAMPT ameliorates disease severity in experimental colitis (32). Its prominent performance in our analysis suggests that metabolic–inflammatory coupling may drive pyroptosis-related activity, particularly in neutrophils. In contrast, PPIF, a key regulator of the mitochondrial permeability transition pore (33), is more closely associated with mitochondrial stress and necrotic cell death (34) rather than GSDMD-mediated pyroptotic execution. LITAF, representing the inflammatory transcriptional regulatory axis, is a critical regulator of TNF-α with established pro-inflammatory roles in IBD (35). Although direct evidence linking LITAF to pyroptosis execution is lacking, its amplification of TNF-α pathways may sensitize upstream inflammasome signaling. Finally, PHACTR1 has relatively weak evidence in the context of IBD, and no studies have yet linked it to either IBD pathogenesis or pyroptosis.

Our single-cell analysis revealed an increase in pyroptosis-related gene signatures within intestinal epithelial cells in IBD. This elevation, however, is most likely reflective of inflammation-induced transcriptional activation and epithelial restitution demands rather than bona fide execution of canonical GSDMD-dependent pyroptosis, as supported by recent mechanistic studies (5). Notably, although neutrophil pyroptosis has been implicated in other inflammatory disorders (36, 37), evidence in the context of IBD has been lacking. Our data provide the first indication that neutrophils exhibit markedly elevated pyroptosis-associated signatures in IBD, suggesting a previously underestimated contribution of neutrophil-driven inflammatory cell death pathways to mucosal inflammation. In line with prior literature, macrophages remain the major immune cell population with established pyroptotic activation in IBD (24). Together, these findings delineate distinct cell-type-specific patterns of pyroptosis-related responses and highlight neutrophils as a potentially overlooked contributor to intestinal inflammation.

Several methodological and interpretative limitations should be acknowledged. First, all analyses were performed using publicly available scRNA-seq and bulk transcriptomic datasets, which may introduce unavoidable batch effects and sample heterogeneity (disease activity, duration, treatment history, intestinal location) despite rigorous quality control. The merging of UC and CD patients into a single “IBD group” for analysis risks obscuring important biological differences between subtypes. Second, comparisons between UC and CD were based on a relatively small number of biological samples (n = 6 per group) and should therefore be interpreted as exploratory. Although batch effects were mitigated using Harmony, subtype-specific differences may still be influenced by sampling variability or unmeasured confounding factors. Importantly, the primary conclusions of this study do not rely on direct UC–CD contrasts. Third, pyroptosis activation was inferred from transcriptional signatures rather than direct functional assays, and therefore may reflect inflammatory gene programs rather than bona fide gasdermin-mediated cell death. Fourth, although multiple machine learning algorithms were integrated to enhance robustness, the diagnostic assessment of candidate genes may still be influenced by dataset-specific bias. The reported AUC values were derived from internal evaluation within a single peripheral blood cohort and should therefore be interpreted as indicative of diagnostic potential rather than definitive performance, warranting further validation in independent cohorts. Finally, the lack of *in vitro* and *in vivo* experiments limits mechanistic interpretation, and future studies are needed to experimentally confirm the regulatory roles of the identified genes in IBD-related pyroptosis.

## Data Availability

The original contributions presented in the study are included in the article/**Supplementary Material**. Further inquiries can be directed to the corresponding authors.
