# Supplementary figures and images for: Integrated single-cell and transcriptomic profiling identifies machine-learning–based pyroptosis biomarkers in IBD
Source: Front Immunol. 2026 Feb 4;17:1761476. doi: 10.3389/fimmu.2026.1761476 (PMC12913491; doi:10.3389/fimmu.2026.1761476)

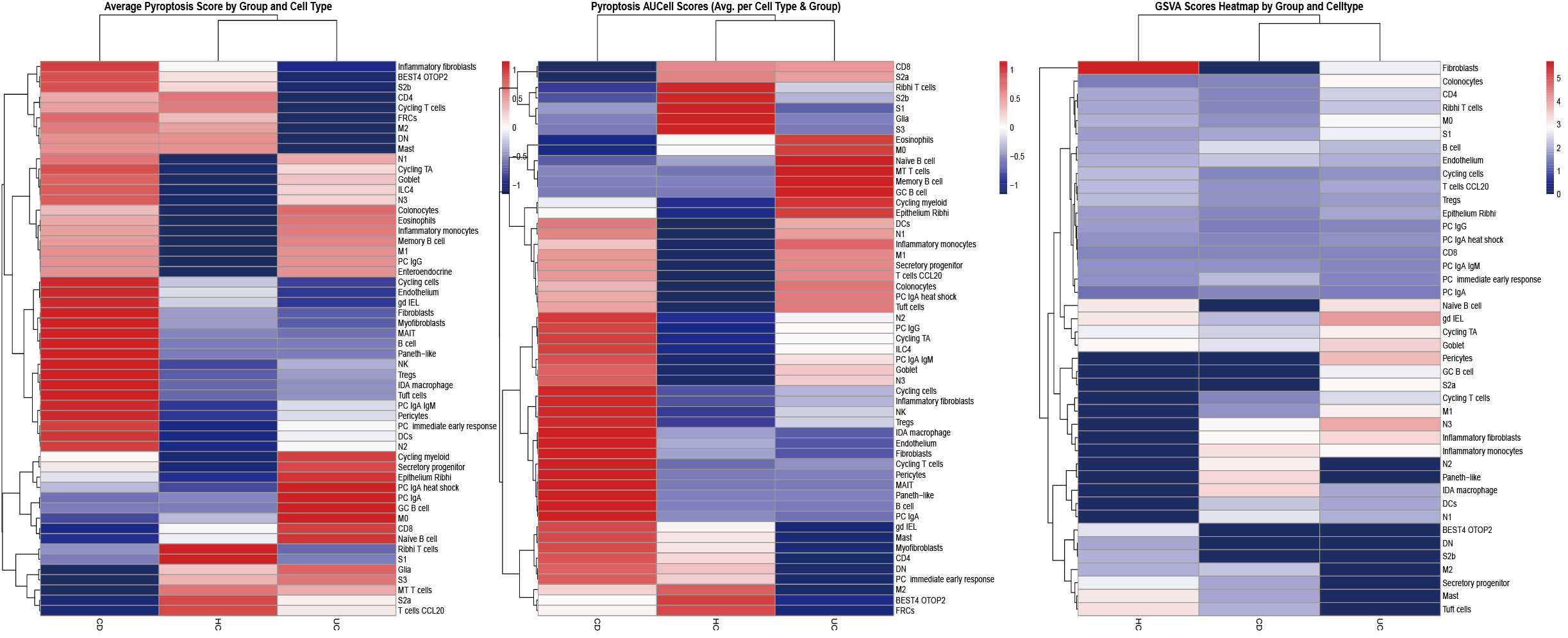

Supplement: Supplementary file 1 [file Image1.tif]
